# The genome sequence of the Small Red Damselfly, *Ceriagrion tenellum *(de Villers, 1789)

**DOI:** 10.12688/wellcomeopenres.23708.1

**Published:** 2025-02-19

**Authors:** Olga Sivell, Duncan Sivell, Ryan Mitchell, Judy Webb, Natural History Museum Genome Acquisition Lab, Darwin Tree of Life Barcoding collective, Wellcome Sanger Institute Tree of Life Management, Samples and Laboratory team, Wellcome Sanger Institute Scientific Operations: Sequencing Operations, Wellcome Sanger Institute Tree of Life Core Informatics team, Tree of Life Core Informatics collective, Darwin Tree of Life Consortium

**Affiliations:** 1 Natural History Museum, London, England, UK; 2 Independent researcher, Sligo, County Sligo, Ireland; 3 Ecological Consultant, Kidlington, England, UK

**Keywords:** Ceriagrion tenellum, Small Red Damselfly, genome sequence, chromosomal, Odonata

## Abstract

We present a genome assembly from a male specimen of *Ceriagrion tenellum* (Small Red Damselfly; Arthropoda; Insecta; Odonata; Coenagrionidae). The genome sequence has a total length of 2,077.00 megabases. Most of the assembly (99.28%) is scaffolded into 14 chromosomal pseudomolecules, including the X sex chromosome. The mitochondrial genome has also been assembled and is 17.21 kilobases in length.

## Species taxonomy

Eukaryota; Opisthokonta; Metazoa; Eumetazoa; Bilateria; Protostomia; Ecdysozoa; Panarthropoda; Arthropoda; Mandibulata; Pancrustacea; Hexapoda; Insecta; Dicondylia; Pterygota; Palaeoptera; Odonata; Zygoptera; Coenagrionoidea; Coenagrionidae; *Ceriagrion*; *Ceriagrion tenellum* (de Villers, 1789) (NCBI:txid638464)

## Background

*Ceriagrion tenellum* is one of two red species of damselflies (Odonata, Zygoptera) and the only one in genus *Ceriagrion* Selys, 1876 occurring in Britain. It can be separated from the common and widespread *Pyrrhosoma nymphula* using the colour of the legs and the wing spot (stigma) – black in *P. nymphula*, reddish in *C. tenellum*. The shoulder stripes are pale and inconspicuous and side of the thorax is yellow with two black stripes. The male abdomen is entirely red (with black markings on S7–9 in *P. nymphula*). The females have three colour forms: *erytrogastrum* (uncommon), which is entirely red, similar to the male; *typica* (most common), mostly black/dark bronze with S1–3 and S9–10 red; and *melanogastrum* (fairly common), with almost entirely dark abdomen with pale segment divisions some of which are reddish.

The larvae of *C. tenellum* are aquatic and feed on insect larvae and other invertebrates in bog-mosses, plants and debris. The larvae are long and thin, 16–17 mm long, brownish, with a relatively short abdomen, with three short, tapering caudal lamellae (“tails”), with prominent veins and no hairs on the distal half ( Hope, 2009; Smallshire & Swash, 2018).

Following copulation, the male guards the female during oviposition in “Agrion” (sentinel) position and they can fly in tandem. The eggs hatch after a month and the larval stage lasts two years. This species spends the last winter before emergence in a late stadium of development, reaching the final larval stadium in spring and proceeding to metamorphosis in the summer. Odonata undergo incomplete metamorphosis, i.e. there is no pupal stage. The final larva crawls out of the water, hardens and an adult emerges from the moult. The flight period is from May to September, with a peak in June and July ( Corbet & Brooks, 2008; Dijkstra, 2006; Dijkstra & Schröter, 2020; Hope, 2009). The species is often found parasitised by water mites, however they have no significant impact on the damselfly’s survivorship, but it could reduce male mating success ( Andres & Cordero, 1998). *C. tenellum* is also a host of the terrestrial mite *Leptus killingtoni*, which is associated with a variety of insects and arachnids ( Kerry, 2013).

This species is listed as Nationally Scarce on the Odonata Red List 2008. It has a local distribution in southern England and western Wales and a small area in Norfolk, in heathlands ( Dijkstra, 2006; NBN Atlas Partnership, 2024; Smallshire & Swash, 2018). *C. tenellum* breeds in acid bog-pools. The distribution of this species became more localised following the drainage and pollution of breeding sites due to agricultural activities, peat extraction, forestry and urban developments, particularly in 1950s and 1960s. It was previously widespread in East Anglia, Dorset and also occurred in the Somerset Levels ( Brooks, 2001; Hope, 2009). Widespread in Europe, in the western Mediterranean, locally common in north-western Europe, also recorded from Croatia, Albania, Slovenia and Crete ( Dijkstra, 2006).

The phylogeny of European Zygoptera was studied by Dijkstra and Kalkman (2012) and Dijkstra *et al*. (2014). The high-quality genome of *Ceriagrion tenellum* presented here was sequenced from a male specimen (NHMUK014036824; SAMEA14448441) from Cothill Fen Nature Reserve, England. The genome was sequenced as part of the Darwin Tree of Life Project, a collaborative effort to sequence all named eukaryotic species in the Atlantic Archipelago of Britain and Ireland. It will aid research on phylogeny and taxonomy of Zygoptera.

## Genome sequence report

**Figure 1.**
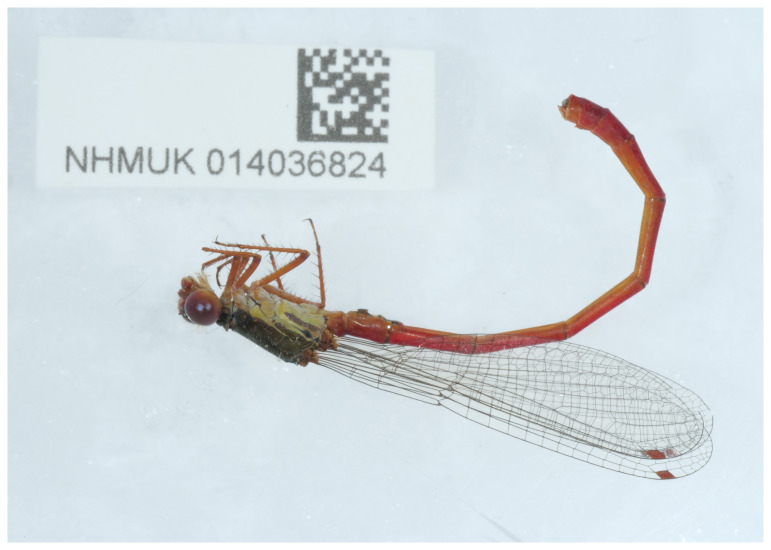
Photograph of the *Ceriagrion tenellum* (ioCerTene1) specimen used for genome sequencing.

The genome of *Ceriagrion tenellum* ( Figure 1) was sequenced using Pacific Biosciences single-molecule HiFi long reads, generating a total of 102.88 Gb (gigabases) from 12.35 million reads, providing an estimated 50-fold coverage. Chromosome conformation Hi-C sequencing produced 155.64 Gb from 1,030.73 million reads. Specimen and sequencing details are summarised in Table 1.

**Table 1.** Specimen and sequencing data for *Ceriagrion tenellum*.

Project information			
**Study title**	Ceriagrion tenellum		
**Umbrella BioProject**	PRJEB62052		
**Species**	*Ceriagrion tenellum*		
**BioSample**	SAMEA14448441		
**NCBI taxonomy ID**	638464		
Specimen information			
**Technology**	**ToLID**	**BioSample accession**	**Organism part**
**PacBio long read sequencing**	ioCerTene1	SAMEA14448867	thorax
**Hi-C sequencing**	ioCerTene1	SAMEA14448866	head
Sequencing information			
**Platform**	**Run accession**	**Read count**	**Base count (Gb)**
**Hi-C Illumina NovaSeq 6000**	ERR11439612	1.03e+09	155.64
**PacBio Sequel IIe**	ERR11458802	2.60e+06	20.27
**PacBio Revio**	ERR11809132	7.13e+06	57.08
**PacBio Sequel IIe**	ERR11458801	2.62e+06	25.53

Assembly errors were corrected by manual curation, including 56 missing joins or mis-joins and 11 haplotypic duplications. This reduced the scaffold number by 7.84%. The final assembly has a total length of 2,077.00 Mb in 469 sequence scaffolds, with 912 gaps, and a scaffold N50 of 152.5 Mb ( Table 2).

**Table 2.** Genome assembly data for *Ceriagrion tenellum*, ioCerTene1.1.

Genome assembly		
Assembly name	ioCerTene1.1	
Assembly accession	GCA_963169105.1	
*Accession of alternate haplotype*	*GCA_963170015.1*	
Span (Mb)	2,077.00	
Number of contigs	1,382	
Number of scaffolds	469	
Longest scaffold (Mb)	201.92	
Assembly metrics *		*Benchmark*
Contig N50 length (Mb)	3.9	*≥ 1 Mb*
Scaffold N50 length (Mb)	152.5	*= chromosome N50*
Consensus quality (QV)	63.4	*≥ 40*
*k*-mer completeness	Primary: 95.16%; alternate: 46.74%; combined: 99.77%	*≥ 95%*
BUSCO **	C:97.0%[S:96.0%,D:1.0%], F:1.7%,M:1.3%,n:1,367	*S > 90%, D < 5%*
Percentage of assembly mapped to chromosomes	99.28%	*≥ 90%*
Sex chromosomes	X	*localised homologous pairs*
Organelles	Mitochondrial genome: 17.21 kb	*complete single alleles*

* Assembly metric benchmarks are adapted from Rhie *et al.* (2021) and the Earth BioGenome Project Report on Assembly Standards September 2024. ** BUSCO scores based on the insecta_odb10 BUSCO set using version 5.4.3. C = complete [S = single copy, D = duplicated], F = fragmented, M = missing, n = number of orthologues in comparison. A full set of BUSCO scores is available at https://blobtoolkit.genomehubs.org/view/Ceriagrion%20tenellum/dataset/CAUJGU01/busco.

**Figure 2.**
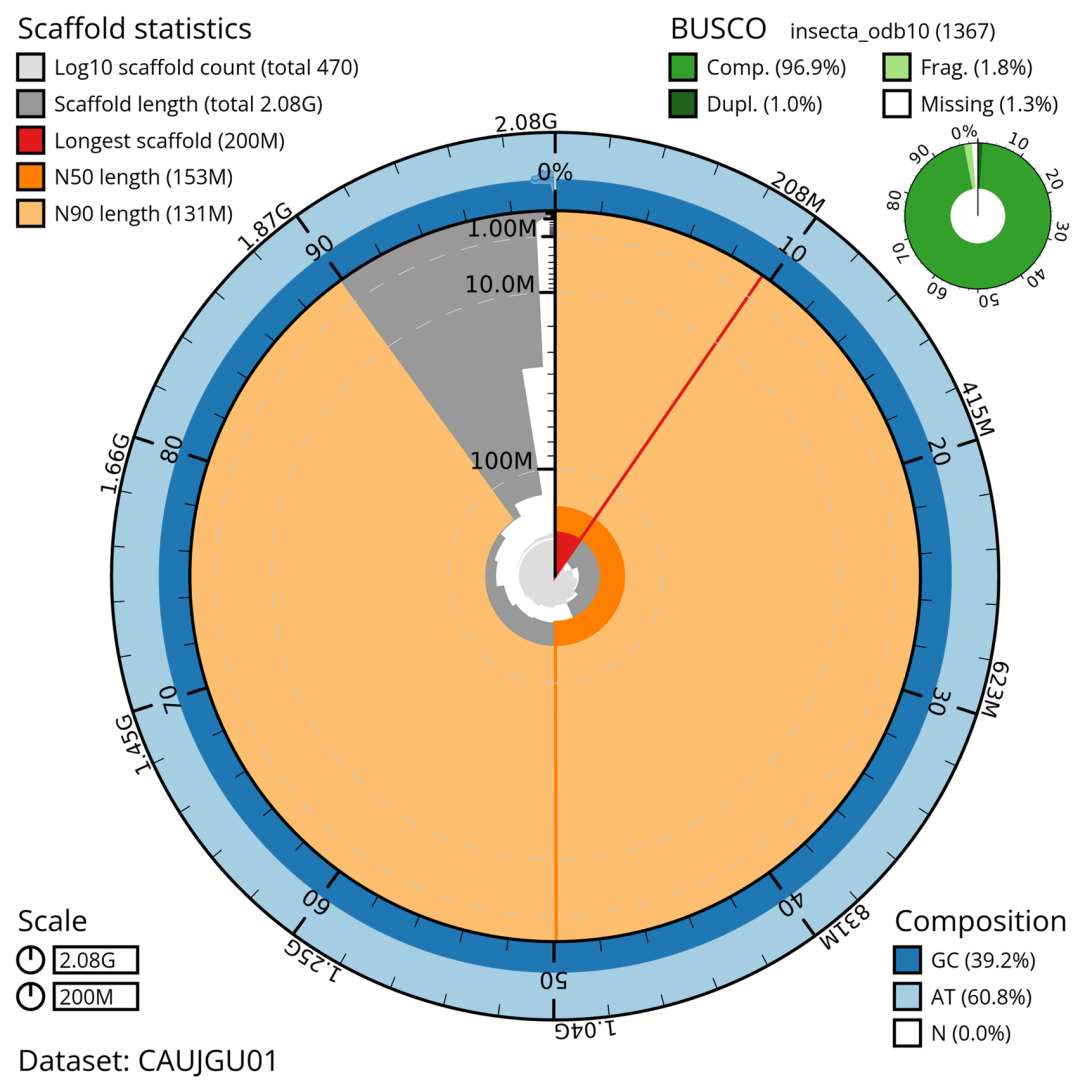
Genome assembly of *Ceriagrion tenellum*, ioCerTene1.1: metrics. The BlobToolKit snail plot provides an overview of assembly metrics and BUSCO gene completeness. The circumference represents the length of the whole genome sequence, and the main plot is divided into 1,000 bins around the circumference. The outermost blue tracks display the distribution of GC, AT, and N percentages across the bins. Scaffolds are arranged clockwise from longest to shortest and are depicted in dark grey. The longest scaffold is indicated by the red arc, and the deeper orange and pale orange arcs represent the N50 and N90 lengths. A light grey spiral at the centre shows the cumulative scaffold count on a logarithmic scale. A summary of complete, fragmented, duplicated, and missing BUSCO genes in the insecta_odb10 set is presented at the top right. An interactive version of this figure is available at https://blobtoolkit.genomehubs.org/view/CAUJGU01/dataset/CAUJGU01/snail.

**Figure 3.**
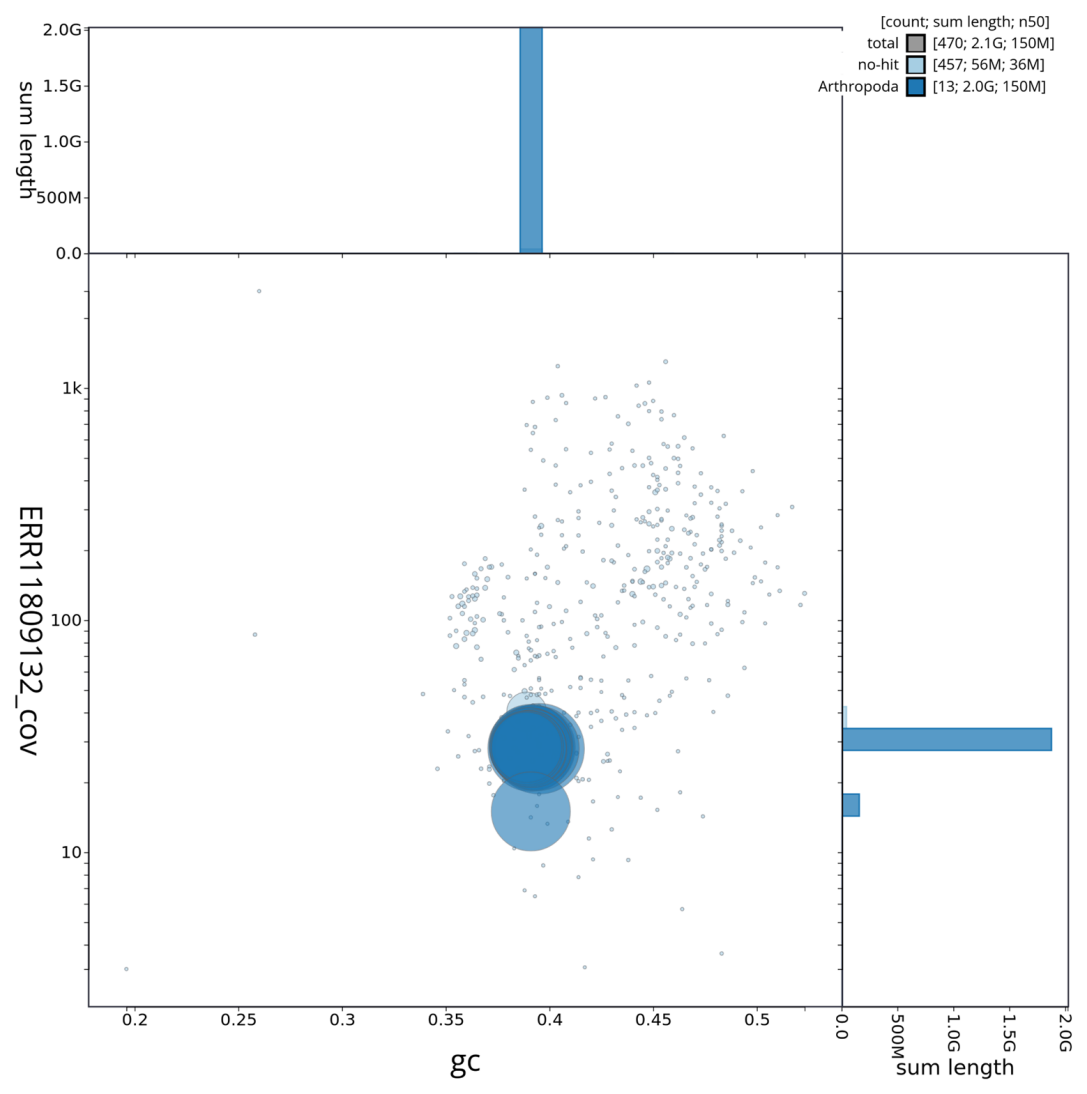
Genome assembly of *Ceriagrion tenellum*, ioCerTene1.1: BlobToolKit GC-coverage plot. Sequences are coloured by phylum. Circles are sized in proportion to sequence length. Histograms show the distribution of sequence length sum along each axis. An interactive version of this figure is available at https://blobtoolkit.genomehubs.org/view/CAUJGU01/dataset/CAUJGU01/blob.

**Figure 4.**
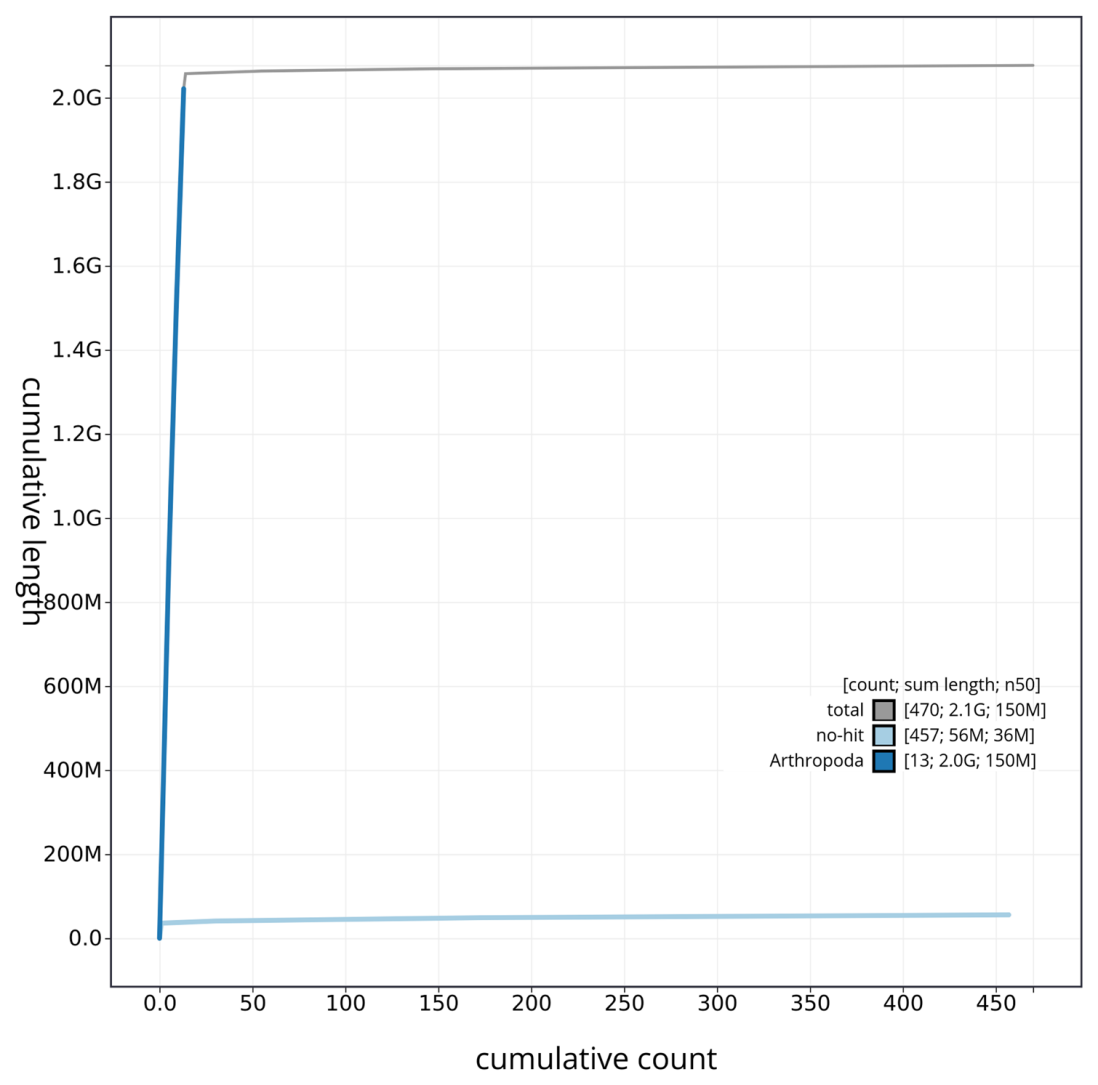
Genome assembly of *Ceriagrion tenellum* ioCerTene1.1: BlobToolKit cumulative sequence plot. The grey line shows cumulative length for all sequences. Coloured lines show cumulative lengths of sequences assigned to each phylum using the buscogenes taxrule. An interactive version of this figure is available at https://blobtoolkit.genomehubs.org/view/CAUJGU01/dataset/CAUJGU01/cumulative.

The snail plot in Figure 2 provides a summary of the assembly statistics, indicating the distribution of scaffold lengths and other assembly metrics. Figure 3 shows the distribution of scaffolds by GC proportion and coverage. Figure 4 presents a cumulative assembly plot, with separate curves representing different scaffold subsets assigned to various phyla, illustrating the completeness of the assembly.

**Figure 5.**
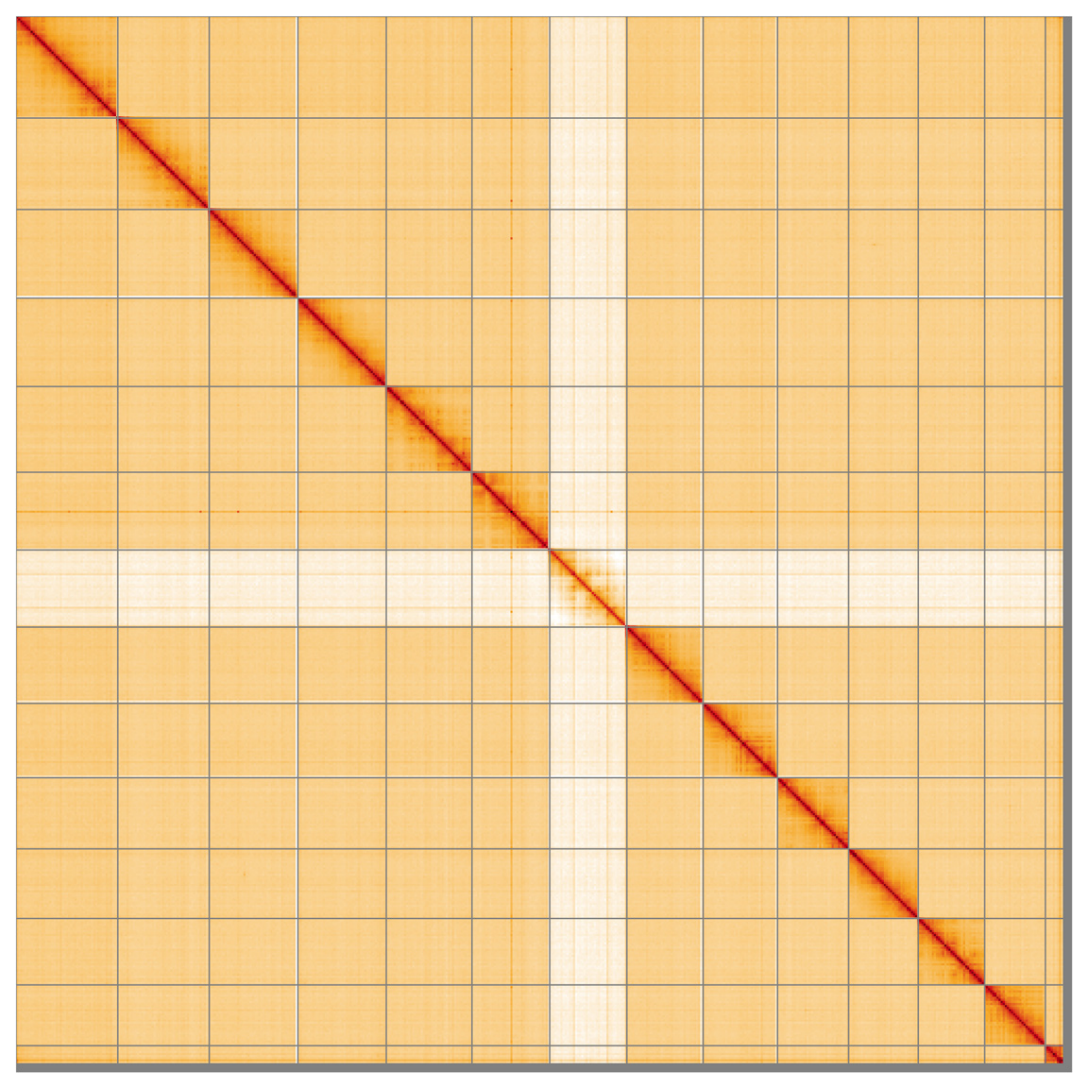
Genome assembly of *Ceriagrion tenellum* ioCerTene1.1: Hi-C contact map of the ioCerTene1.1 assembly, visualised using HiGlass. Chromosomes are shown in order of size from left to right and top to bottom. An interactive version of this figure may be viewed at https://genome-note-higlass.tol.sanger.ac.uk/l/?d=B0Vvca0zRQu64jEfjntMcw.

Most of the assembly sequence (99.28%) was assigned to 14 chromosomal-level scaffolds, representing 13 autosomes and the X sex chromosome. These chromosome-level scaffolds, confirmed by the Hi-C data, are named in order of size ( Figure 5; Table 3). While not fully phased, the assembly deposited is of one haplotype. Contigs corresponding to an alternate haplotype have also been deposited.

**Table 3.** Chromosomal pseudomolecules in the genome assembly of *Ceriagrion tenellum*, ioCerTene1.

INSDC accession	Name	Length (Mb)	GC%
OY720632.1	1	199.9	39.5
OY720633.1	2	179.75	39.0
OY720634.1	3	173.82	39.0
OY720635.1	4	173.51	39.5
OY720636.1	5	168.51	39.0
OY720637.1	6	152.52	39.0
OY720639.1	7	149.87	39.0
OY720640.1	8	146.2	39.0
OY720641.1	9	139.5	39.0
OY720642.1	10	136.49	39.0
OY720643.1	11	130.71	39.0
OY720644.1	12	119.16	39.0
OY720645.1	13	35.93	39.0
OY720638.1	X	151.36	39.0
OY720646.1	MT	0.02	26.0

The mitochondrial genome was also assembled and can be found as a contig within the multifasta file of the genome submission, and as a separate fasta file.

The final assembly has a Quality Value (QV) of 63.4. The *k*-mer completeness was 95.16% for the primary assembly, 46.74% for the alternate haplotype and 99.77% for the combined assemblies. BUSCO (v5.4.3) analysis using the insecta_odb10 reference set ( *n* = 1,367) indicated a completeness score of 97.0% (single = 96.0%, duplicated = 1.0%). The assembly achieves the Earth Biogenome Project (EBP) reference standard of **6.C.Q63**. Other quality metrics are given in Table 2.

## Methods

### Sample acquisition and DNA barcoding

A male adult *Ceriagrion tenellum* (specimen ID NHMUK014036824, ToLID ioCerTene1) was collected from Cothill Fen Nature Reserve, England, United Kingdom (latitude 51.69, longitude –1.33) on 2021-06-19 by aerial net. The specimen was collected by Olga Sivell, Duncan Sivell, Ryan Mitchell, Judy Webb, then identified by Judy Webb and preserved in ethanol.

The initial identification was verified by an additional DNA barcoding process according to the framework developed by Twyford *et al*. (2024). A small sample was dissected from the specimens and stored in ethanol, while the remaining parts were shipped on dry ice to the Wellcome Sanger Institute (WSI) ( Pereira *et al.*, 2022). The tissue was lysed, the COI marker region was amplified by PCR, and amplicons were sequenced and compared to the BOLD database, confirming the species identification ( Crowley *et al.*, 2023). Following whole genome sequence generation, the relevant DNA barcode region was also used alongside the initial barcoding data for sample tracking at the WSI ( Twyford *et al.*, 2024). The standard operating procedures for Darwin Tree of Life barcoding have been deposited on protocols.io ( Beasley *et al.*, 2023).

### Nucleic acid extraction

The workflow for high molecular weight (HMW) DNA extraction at the Wellcome Sanger Institute (WSI) Tree of Life Core Laboratory includes a sequence of procedures: sample preparation and homogenisation, DNA extraction, fragmentation and purification. Detailed protocols are available on protocols.io ( Denton *et al.*, 2023b).

The ioCerTene1 sample was prepared for DNA extraction by weighing and dissecting it on dry ice ( Jay *et al.*, 2023). Tissue from the thorax was homogenised using a PowerMasher II tissue disruptor ( Denton *et al.*, 2023a). HMW DNA was extracted in the WSI Scientific Operations core using the Automated MagAttract v2 protocol ( Oatley *et al.*, 2023). The DNA was sheared into an average fragment size of 12–20 kb in a Megaruptor 3 system ( Bates *et al.*, 2023). Sheared DNA was purified by solid-phase reversible immobilisation, using AMPure PB beads to eliminate shorter fragments and concentrate the DNA ( Strickland *et al.*, 2023). The concentration of the sheared and purified DNA was assessed using a Nanodrop spectrophotometer and Qubit Fluorometer using the Qubit dsDNA High Sensitivity Assay kit. Fragment size distribution was evaluated by running the sample on the FemtoPulse system.

### Hi-C sample preparation

Tissue from the head of the ioCerTene1 sample was processed at the WSI Scientific Operations core, using the Arima-HiC v2 kit. In brief, 20–50 mg of frozen tissue (stored at –80 °C) was fixed, and the DNA crosslinked using a TC buffer with 22% formaldehyde concentration. After crosslinking, the tissue was homogenised using the Diagnocine Power Masher-II and BioMasher-II tubes and pestles. Following the Arima-HiC v2 kit manufacturer's instructions, crosslinked DNA was digested using a restriction enzyme master mix. The 5’-overhangs were filled in and labelled with biotinylated nucleotides and proximally ligated. An overnight incubation was carried out for enzymes to digest remaining proteins and for crosslinks to reverse. A clean up was performed with SPRIselect beads prior to library preparation. Additionally, the biotinylation percentage was estimated using the Qubit Fluorometer v4.0 (Thermo Fisher Scientific) and Qubit HS Assay Kit and Arima-HiC v2 QC beads.

### Library preparation and sequencing

At a minimum, samples were required to have an average fragment size exceeding 8 kb and a total mass over 400 ng to proceed to the low input SMRTbell Prep Kit 3.0 protocol (Pacific Biosciences, California, USA), depending on genome size and sequencing depth required. Libraries were prepared using the SMRTbell Prep Kit 3.0 (Pacific Biosciences, California, USA) as per the manufacturer's instructions. The kit includes the reagents required for end repair/A-tailing, adapter ligation, post-ligation SMRTbell bead cleanup, and nuclease treatment. Following the manufacturer’s instructions, size selection and clean up was carried out using diluted AMPure PB beads (Pacific Biosciences, California, USA). DNA concentration was quantified using the Qubit Fluorometer v4.0 (Thermo Fisher Scientific) with Qubit 1X dsDNA HS assay kit and the final library fragment size analysis was carried out using the Agilent Femto Pulse Automated Pulsed Field CE Instrument (Agilent Technologies) and gDNA 55kb BAC analysis kit.

Samples were sequenced using the Sequel IIe system (Pacific Biosciences, California, USA). The concentration of the library loaded onto the Sequel IIe was in the range 40–135 pM. The SMRT link software, a PacBio web-based end-to-end workflow manager, was used to set-up and monitor the run, as well as perform primary and secondary analysis of the data upon completion.

Samples were also sequenced on a Revio instrument (Pacific Biosciences, California, USA). Prepared libraries were normalised to 2 nM, and 15 μL was used for making complexes. Primers were annealed and polymerases were hybridised to create circularised complexes according to manufacturer’s instructions. The complexes were purified with the 1.2X clean up with SMRTbell beads. The purified complexes were then diluted to the Revio loading concentration (in the range 200–300 pM), and spiked with a Revio sequencing internal control. Samples were sequenced on Revio 25M SMRT cells (Pacific Biosciences, California, USA). The SMRT link software, a PacBio web-based end-to-end workflow manager, was used to set-up and monitor the run, as well as perform primary and secondary analysis of the data upon completion.

***Hi-C***

For Hi-C library preparation, DNA was fragmented using the Covaris E220 sonicator (Covaris) and size selected using SPRISelect beads to 400 to 600 bp. The DNA was then enriched using the Arima-HiC v2 kit Enrichment beads. Using the NEBNext Ultra II DNA Library Prep Kit (New England Biolabs) for end repair, a-tailing, and adapter ligation. This uses a custom protocol which resembles the standard NEBNext Ultra II DNA Library Prep protocol but where library preparation occurs while DNA is bound to the Enrichment beads. For library amplification, 10 to 16 PCR cycles were required, determined by the sample biotinylation percentage. The Hi-C sequencing was performed using paired-end sequencing with a read length of 150 bp on an Illumina NovaSeq 6000 instrument.

### Genome assembly, curation and evaluation

***Assembly***

The HiFi reads were first assembled using Hifiasm ( Cheng *et al.*, 2021) with the --primary option. Haplotypic duplications were identified and removed using purge_dups ( Guan *et al.*, 2020). The Hi-C reads were mapped to the primary contigs using bwa-mem2 ( Vasimuddin *et al.*, 2019). The contigs were further scaffolded using the provided Hi-C data ( Rao *et al.*, 2014) in YaHS ( Zhou *et al.*, 2023) using the --break option for handling potential misassemblies. The scaffolded assemblies were evaluated using Gfastats ( Formenti *et al.*, 2022), BUSCO ( Manni *et al.*, 2021) and MERQURY.FK ( Rhie *et al.*, 2020).

The mitochondrial genome was assembled using MitoHiFi ( Uliano-Silva *et al.*, 2023), which runs MitoFinder ( Allio *et al.*, 2020) and uses these annotations to select the final mitochondrial contig and to ensure the general quality of the sequence.

***Assembly curation***

The assembly was decontaminated using the Assembly Screen for Cobionts and Contaminants (ASCC) pipeline (article in preparation). Manual curation was primarily conducted using PretextView ( Harry, 2022), with additional insights provided by JBrowse2 ( Diesh *et al.*, 2023) and HiGlass ( Kerpedjiev *et al.*, 2018). Scaffolds were visually inspected and corrected as described by Howe *et al*. (2021). Any identified contamination, missed joins, and mis-joins were corrected, and duplicate sequences were tagged and removed. The curation process is documented at https://gitlab.com/wtsi-grit/rapid-curation (article in preparation).

***Assembly quality assessment***

The Merqury.FK tool ( Rhie *et al.*, 2020), run in a Singularity container ( Kurtzer *et al.*, 2017), was used to evaluate *k*-mer completeness and assembly quality for the primary and alternate haplotypes using the *k*-mer databases ( *k* = 31) that were computed prior to genome assembly. The analysis outputs included assembly QV scores and completeness statistics.

A Hi-C contact map was produced for the final version of the assembly. The Hi-C reads were aligned using bwa-mem2 ( Vasimuddin *et al.*, 2019) and the alignment files were combined using SAMtools ( Danecek *et al.*, 2021). The Hi-C alignments were converted into a contact map using BEDTools ( Quinlan & Hall, 2010) and the Cooler tool suite ( Abdennur & Mirny, 2020). The contact map is visualised in HiGlass ( Kerpedjiev *et al.*, 2018).

The genome was also analysed within the BlobToolKit environment ( Challis *et al.*, 2020) and BUSCO scores ( Manni *et al.*, 2021) were calculated.

Table 4 contains a list of relevant software tool versions and sources.

**Table 4.** Software tools: versions and sources.

Software tool	Version	Source
BEDTools	2.30.0	https://github.com/arq5x/bedtools2
BLAST	2.14.0	ftp://ftp.ncbi.nlm.nih.gov/blast/executables/blast+/
BlobToolKit	4.3.7	https://github.com/blobtoolkit/blobtoolkit
BUSCO	5.3.2	https://gitlab.com/ezlab/busco
bwa-mem2	2.2.1	https://github.com/bwa-mem2/bwa-mem2
Cooler	0.8.11	https://github.com/open2c/cooler
fasta_windows	0.2.4	https://github.com/tolkit/fasta_windows
FastK	427104ea91c78c3b8b8b49f1a7d6bbeaa869ba1c	https://github.com/thegenemyers/FASTK
Gfastats	1.3.6	https://github.com/vgl-hub/gfastats
Hifiasm	0.19.8-r587	https://github.com/chhylp123/hifiasm
HiGlass	44086069ee7d4d3f6f3f0012569789ec138f42b84a a44357826c0b6753eb28de	https://github.com/higlass/higlass
Merqury.FK	d00d98157618f4e8d1a9190026b19b471055b22e	https://github.com/thegenemyers/MERQURY.FK
MitoHiFi	3	https://github.com/marcelauliano/MitoHiFi
Nextflow	23.04.0-5857	https://github.com/nextflow-io/nextflow
PretextView	0.2.5	https://github.com/sanger-tol/PretextView
purge_dups	1.2.5	https://github.com/dfguan/purge_dups
samtools	1.16.1, 1.17, and 1.18	https://github.com/samtools/samtools
sanger-tol/ascc	-	https://github.com/sanger-tol/ascc
Singularity	3.9.0	https://github.com/sylabs/singularity
YaHS	1.2a.2	https://github.com/c-zhou/yahs

### Wellcome Sanger Institute – Legal and Governance

The materials that have contributed to this genome note have been supplied by a Darwin Tree of Life Partner. The submission of materials by a Darwin Tree of Life Partner is subject to the **‘Darwin Tree of Life Project Sampling Code of Practice’**, which can be found in full on the Darwin Tree of Life website here. By agreeing with and signing up to the Sampling Code of Practice, the Darwin Tree of Life Partner agrees they will meet the legal and ethical requirements and standards set out within this document in respect of all samples acquired for, and supplied to, the Darwin Tree of Life Project.

Further, the Wellcome Sanger Institute employs a process whereby due diligence is carried out proportionate to the nature of the materials themselves, and the circumstances under which they have been/are to be collected and provided for use. The purpose of this is to address and mitigate any potential legal and/or ethical implications of receipt and use of the materials as part of the research project, and to ensure that in doing so we align with best practice wherever possible. The overarching areas of consideration are:

•   Ethical review of provenance and sourcing of the material

•   Legality of collection, transfer and use (national and international)

Each transfer of samples is further undertaken according to a Research Collaboration Agreement or Material Transfer Agreement entered into by the Darwin Tree of Life Partner, Genome Research Limited (operating as the Wellcome Sanger Institute), and in some circumstances other Darwin Tree of Life collaborators.

## Data Availability

European Nucleotide Archive: Ceriagrion tenellum. Accession number PRJEB62052; https://identifiers.org/ena.embl/PRJEB62052. The genome sequence is released openly for reuse. The *Ceriagrion tenellum* genome sequencing initiative is part of the Darwin Tree of Life (DToL) project. All raw sequence data and the assembly have been deposited in INSDC databases. The genome will be annotated using available RNA-Seq data and presented through the Ensembl pipeline at the European Bioinformatics Institute. Raw data and assembly accession identifiers are reported in Table 1 and Table 2.

## Author information

Members of the Natural History Museum Genome Acquisition Lab are listed here: https://doi.org/10.5281/zenodo.12159242.

Members of the Darwin Tree of Life Barcoding collective are listed here: https://doi.org/10.5281/zenodo.12158331.

Members of the Wellcome Sanger Institute Tree of Life Management, Samples and Laboratory team are listed here: https://doi.org/10.5281/zenodo.12162482.

Members of Wellcome Sanger Institute Scientific Operations: Sequencing Operations are listed here: https://doi.org/10.5281/zenodo.12165051.

Members of the Wellcome Sanger Institute Tree of Life Core Informatics team are listed here: https://doi.org/10.5281/zenodo.12160324.

Members of the Tree of Life Core Informatics collective are listed here: https://doi.org/10.5281/zenodo.12205391.

Members of the Darwin Tree of Life Consortium are listed here: https://doi.org/10.5281/zenodo.4783558.
