# Supplementary material for: The genome sequence of the Small Red Damselfly, Ceriagrion tenellum (de Villers, 1789)
Source: Wellcome Open Res. 2025 Feb 19;10:79. [Version 1] doi: 10.12688/wellcomeopenres.23708.1 (PMC11911719; doi:10.12688/wellcomeopenres.23708.1)
Supplement: Peer review history

# Reviewer report: Reviewer response for version 1

**DOI:** 10.21956/wellcomeopenres.26156.r119837

**Published:** 2025-03-16

**Authors:** Panagiotis Ioannidis

**Affiliations:** 1 Foundation for Research & Technology - Hellas, Crete, Greece

I'm happy to see that one more Odonate genome has been sequenced! The manuscript gives a very nice overview of the biology of this insect. Additionally, all the submitted data look good and I hope that they will be used by the respective scientific community in order to advance research on this very important group of insects.

 My only suggestion to the authors is to consider using the term "naiads" for describing the nymphal stage of *C. tenellum* (it's common for nymphs of aquatic insects).

Are sufficient details of methods and materials provided to allow replication by others?

Yes

Is the rationale for creating the dataset(s) clearly described?

Yes

Are the datasets clearly presented in a useable and accessible format?

Yes

Are the protocols appropriate and is the work technically sound?

Yes

Reviewer Expertise:

Bioinformatics, Insect genomics

I confirm that I have read this submission and believe that I have an appropriate level of expertise to confirm that it is of an acceptable scientific standard.

# Reviewer report: Reviewer response for version 1

**DOI:** 10.21956/wellcomeopenres.26156.r119275

**Published:** 2025-03-11

**Authors:** James B Whitfield

**Affiliations:** 1 University of Illinois at Urbana-Champaign, Champaign, USA

This report summarizes the results of sequencing the genome of the Small Red Damselfly, *Ceriagrion tenellum*, based on a single male specimen. As with many, although not all, early-diverging lineages of insects, the genome is large, more on the size of the human genome than that of most insects. The results will be of interest not only in the context of insect phylogeny, but as a foundation for the underlying genetics of complex courtship behaviour in Odonata.

 The methods are state of the art and clearly described and the genome quality is good, considering the challenges presented by the genome size.

Are sufficient details of methods and materials provided to allow replication by others?

Yes

Is the rationale for creating the dataset(s) clearly described?

Yes

Are the datasets clearly presented in a useable and accessible format?

Yes

Are the protocols appropriate and is the work technically sound?

Yes

Reviewer Expertise:

Insect phylogenetics and comparative study, including genomics

I confirm that I have read this submission and believe that I have an appropriate level of expertise to confirm that it is of an acceptable scientific standard.
